# Review: Enteric Methane Emissions Across Physiological Stages and Dietary NDF/NFC Ratios in Holstein Dairy Cattle—Implications for China’s Carbon Mitigation

**DOI:** 10.3390/ani16111684

**Published:** 2026-05-30

**Authors:** Peng Jia, Yan Tu, Naifeng Zhang, Naisheng Lu, Hulong Lei, Xueyuan Jiang, Qiyu Diao

**Affiliations:** 1 Institute of Animal Husbandry and Veterinary Science, Shanghai Academy of Agricultural Sciences, Shanghai 201106, Chinalunaisheng@saas.sh.cn (N.L.); jiangxueyuan@saas.sh.cn (X.J.); 2 Institute of Feed Research, Chinese Academy of Agricultural Sciences/Sino-US Joint Laboratory on Nutrition and Metabolism of Ruminant/Key Laboratory of Feed Biotechnology of the Ministry of Agriculture and Rural Affairs/Beijing Innovation Consortium of Agriculture Research System, Beijing 100081, China; tuyan@caas.cn (Y.T.);

**Keywords:** Holstein dairy cattle, methane emissions, methane mitigation, physiological stages, NDF/NFC ratios

## Abstract

Dairy cattle are a substantial contributor to global agricultural greenhouse gas emissions, primarily producing enteric methane through the ruminal anaerobic fermentation of dietary fiber. As China formally pledges to achieve carbon neutrality before 2060, accurately quantifying these emissions and developing localized mitigation strategies within the livestock sector has become a critical priority. Enteric methane emissions in dairy cattle are not a static physiological baseline; rather, they represent a highly dynamic phenotype profoundly influenced by an intricate network of physiological and environmental parameters. These include the animal’s age, anatomical and ruminal development, parity, lactation stage, and the precise stoichiometric balance of dietary carbohydrates. This review synthesizes extensive experimental data to construct a robust, scientifically logical framework elucidating the profound physiological mechanisms that govern apparent methane emission parameters. Accordingly, this paper reviews our recent research on methane emissions from Holstein dairy cattle across various ages and lactation stages, including heifers, lactating cows, and dry cows. Furthermore, it extensively evaluates the modulation of methanogenesis under diets with varying neutral detergent fiber to non-fibrous carbohydrate (NDF/NFC) ratios, demonstrating that an increased NDF/NFC ratio is positively correlated with higher enteric methane production, yield, and intensity due to the promotion of acetate-type ruminal fermentation. Ultimately, this review aims to provide robust theoretical support for the accurate quantification of enteric methane emissions and the formulation of precision mitigation strategies tailored to specific physiological states.

## Simple Summary

Reducing greenhouse gas emissions from agriculture is essential for slowing climate change. Dairy cattle are a major source of methane, a gas produced naturally during the digestion of plant-based feeds. This study aims to understand how methane emissions from Holstein dairy cattle change across different ages, physiological stages, and diet compositions. By measuring methane released from growing heifers, lactating cows, and dry cows, the research reveals that lactating cows emit the most methane, while young heifers emit the least. Furthermore, diets higher in fibrous plant materials lead to greater methane production compared to diets rich in easily digestible carbohydrates. These findings demonstrate that methane emissions vary widely based on the animal’s physical state and feed. Understanding these natural variations provides a valuable foundation for accurately estimating national agricultural emissions. Ultimately, these insights will help the agricultural sector design targeted feeding strategies to reduce methane emissions without compromising milk production, thereby supporting global environmental goals.

## 1. Introduction

Ruminants can convert forage and other materials that cannot be used by monogastric animals, such as pigs and poultry, into high-quality meat and milk, which play an irreplaceable role in the human diet. However, after these fibrous feeds enter the rumen, they produce volatile fatty acids, hydrogen, and carbon dioxide through microbial anaerobic fermentation, and methanogens inevitably utilize these byproducts to produce methane [1]. Methane cannot be utilized by the ruminant and is directly excreted through eructation and respiration [2]. This process results in the loss of 2% to 12% of the animal’s total energy intake [3]. Environmentally, although methane is the second most abundant component of greenhouse gases, its warming potential is 28 times that of carbon dioxide (the most abundant component) [4]. Methane remains in the atmosphere for only 12.2 years, which is far less than the 100-year residence time of carbon dioxide [5]. The Intergovernmental Panel on Climate Change (IPCC) emphasized the importance of methane abatement for the first time in 2021 and proposed that rapid and comprehensive control of methane emissions is an effective means to slow the rate of climate warming in the short term [6]. Consequently, reducing methane emissions is the fastest way to mitigate the greenhouse effect.

Although agriculture is widely recognized as a primary source of anthropogenic methane globally, the specific contribution of ruminant enteric fermentation varies considerably across different countries and regions. In the People’s Republic of China, agricultural activities are a major source of greenhouse gases, with enteric fermentation serving as the largest single contributor, accounting for approximately 43.9% of the national agricultural methane emissions [7]. In the United States, enteric fermentation is a dominant methane source, representing 27.4% of the country’s total anthropogenic methane emissions [8]. Similarly, in the European Union, the agricultural sector is responsible for about 56% of all anthropogenic methane, and remarkably, 80% of these agricultural emissions originate directly from enteric fermentation in ruminants [9]. Despite these regional variations in national emission inventories, global estimates consistently demonstrate that livestock contributes to approximately one-third of the total anthropogenic methane emissions worldwide [6]. Therefore, implementing targeted mitigation strategies to reduce enteric methane emissions from ruminants, particularly cattle, remains a universally critical priority for combating global climate change.

As the global population grows and people’s living standards improve, ruminant farming will continue to expand to meet the demand for meat and milk, as a predicted 48% increase in global dairy demand is expected between 2005 and 2050 [10]. If greenhouse gas emissions from the livestock industry are not abated, methane emissions from livestock could increase by 60% by 2030, according to the Food and Agriculture Organization (FAO) of the United Nations [11]. The Third National Communication on Climate Change of the People’s Republic of China pointed out that China’s anthropogenic methane emissions mainly come from animal husbandry, which accounts for 40.5% of the national total [12]. China currently raises more than 6 million dairy cattle, and a methane emission reduction strategy for dairy cattle is necessary to support China’s national carbon emission reduction goals. Methane emissions from ruminants are affected by factors including animal age, physiological stage, and dietary composition [13,14,15]. Diets characterized by a high ratio of neutral detergent fiber (NDF) to non-fibrous carbohydrates (NFC) typically promote acetate-type fermentation, which increases the availability of metabolic hydrogen and subsequently drives greater enteric methane production [16]. Conversely, increasing the dietary proportion of NFC shifts the ruminal microbiome toward propionate-type fermentation, acting as a competitive hydrogen sink that effectively mitigates methane emissions [16]. To develop strategies for methane mitigation without affecting the production performance (such as growth performance in heifers and lactation performance in lactating cows) to meet human needs, knowledge about the methane emission characteristics of these animals is indispensable.

In addition, it should be emphasized that due to differences in animal methane emissions caused by external factors, methane emission factors (EF, kg CH_4_·head^−1^·yr^−1^) of animals in other regions cannot accurately represent those of local animals. For instance, a study published in the journal Nature reported that China’s methane emissions between 2000 and 2015 were overestimated by 15% [17], suggesting that it would be more accurate to use country-specific methane emission factors. We conducted experiments to reveal variations in methane emissions from Chinese dairy cattle at different physiological stages (including heifers, lactating cows, and dry cows), as well as the effect of diets with different neutral detergent fiber (NDF)/non-fibrous carbohydrates (NFC) ratios on methane emissions. These findings help enrich China’s methane emissions database and reduce estimation errors and uncertainties in mitigation strategies [18]. Therefore, this review synthesizes recent empirical advancements evaluating the characteristics of methane emissions from dairy cattle in China to provide a theoretical basis for future studies on ruminant methane emissions and the formulation of greenhouse gas emission reduction targets for the livestock industry.

## 2. Methane Emissions from Holstein Dairy Cattle at Different Physiological Stages

The growth, lactation, and dry periods are three critical physiological stages of dairy cattle; therefore, we targeted methane emissions during these three stages. In order to accurately measure the methane emissions from large herds of dairy cattle housed under modern dairy farm conditions, we used the sulfur hexafluoride tracer technique and the GreenFeed system.

### 2.1. Methane Emissions from Heifers at Different Ages

**Figure 1:**
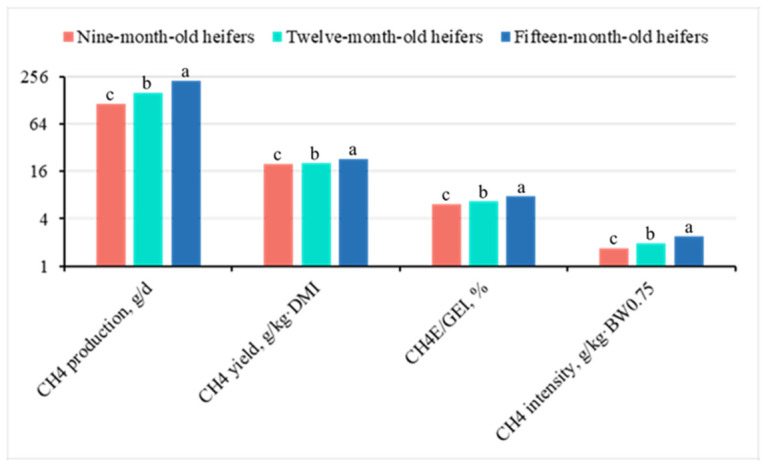
Methane emissions from heifers at different ages. Within the same indicator, different letters indicate significant differences (*p* < 0.05). *n* = 15. CH_4_: methane; DMI: dry matter intake; CH_4_E: methane energy; GEI: gross energy intake; BW^0.75^: metabolic weight.

Heifers are the backup force for adult cows and generally account for nearly half of the total herd on dairy farms. We measured enteric methane emissions in 9-, 12-, and 15-month-old Holstein heifers using the sulfur hexafluoride tracer technique, which provided previously unavailable data on methane emissions from heifers in China. Methane emissions of Holstein heifers were measured utilizing the sulfur hexafluoride tracer technique [19,20]. On the final day of the experimental period, the body weight of the heifers was recorded using an electronic weighbridge prior to the morning feeding. Fifteen heifers in each age group were fed the same diet containing 30% concentrate and 70% forage on a dry matter basis (Table S8). Following a 14-day adaptation period, a 7-day digestion trial and a consecutive six-day methane measurement phase were conducted. During the digestion trial, fecal samples (100 g per sampling) were collected thrice daily (morning, noon, and evening) via rectal grab sampling. The apparent total tract digestibility of nutrients, specifically dry matter, organic matter, crude protein, ether extract, NDF, and acid detergent fiber (ADF), was subsequently calculated utilizing the acid-insoluble ash internal marker method. The results showed that the methane production of heifers increased with age from 117.7 to 229.6 g/d, as did the methane yield (from 19.7 to 23.2 g/kg·DMI), methane intensity (from 1.66 to 2.39 g/kg·BW0.75), and methane energy as a percentage of gross energy intake (from 6.10% to 7.69%) (Figure 1, Table S1).

In heifers, dry matter intake increased with age (from 6.0 to 10.0 kg/d), as did body weight. Feed intake has been shown to be the primary driver of enteric methanogenesis and is positively correlated with methane emissions [21,22,23]. Therefore, the increase in dry matter intake may have directly contributed to the highest methane emissions observed in the 15-month-old heifers. However, even when calculating the methane emissions per kg of dry matter intake, the emissions of growing heifers still tended to increase with age. This may be because the heifer’s digestive system was still developing [24], and as it matured, it led to better digestive function, which in turn provided more hydrogen and carbon dioxide for methanogenesis.

The improvement in the feed intake capacity of dairy cattle with age has been reported in several studies [25,26]. Older heifers have improved chewing behavior, chewing ability and regurgitation ability, and are better able to grind and digest their feed [27,28]. We found that the apparent digestibility of NDF and ADF of 15-month-old heifers was 5.97% and 6.82% higher than that of 12-month-old heifers, respectively. In agreement with our results, an earlier study also reported that the digestibility of dietary nutrients was age-dependent in sheep [29]. The anaerobic fermentation of fibrous diets in the rumen provides substrates such as hydrogen and carbon dioxide for methanogenesis; thus, the change in fiber digestibility with age may also be the reason for the variations in methane emissions in different ages of heifers. Previous research data show that methane production (g/d), methane yield (g/kg·DMI), methane emissions per kg of digestible NDF intake, and methane energy as a percentage of gross energy intake were significantly positively correlated with the age of heifers [30], which is consistent with our findings. In contrast to our results, Grandl et al. [30] demonstrated that methane emissions per kg of body weight from heifers did not increase with age. This discrepancy may be explained by the fact that the heifers in their experiment were fed a forage-only diet, resulting in a dry matter intake relative to body weight that did not increase as the animals aged [30].

### 2.2. Methane Emissions from Lactating Cows at Different Lactation Periods

**Figure 2:**
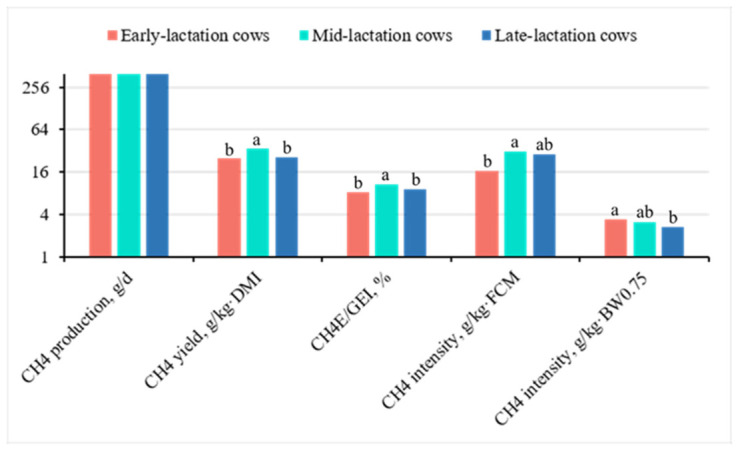
Methane emissions from lactating cows at different lactation periods. Within the same indicator, different letters indicate significant differences (*p* < 0.05). *n* = 12. CH_4_: methane; DMI: dry matter intake; CH_4_E: methane energy; GEI: gross energy intake; FCM: fat-corrected milk; BW^0.75^: metabolic weight.

Milk production is the most important physiological characteristic of Holstein dairy cows; thus, we selected cows in early, mid and late lactation periods (mean ± SD; 88 ± 15.3, 170 ± 19.0, and 243 ± 15.3 of days in milk) as experimental animals. Twelve lactating cows were included in each lactation period. Early, mid, and late lactating cows were fed diets with forage:concentrate ratios of 44:56, 46:54, and 30:70, respectively (Table S9). After a 14-day adaptation period, a three-day digestion test and a six-day methane measurement test were carried out. The digestion trial followed the same methodology as the aforementioned heifer trial. Methane emissions of Holstein heifers were measured utilizing the sulfur hexafluoride tracer technique [19,20]. The measurement of lactational performance in dairy cows was based on the method described by Dong et al. [31]. We found that the methane yield of the lactating Holstein cows was approximately 28.70 g/kg·DMI, methane intensity was 3.02 g/kg·BW0.75 or 25.42 g/kg·FCM, and methane energy as a percentage of gross energy intake was 9.32% (Figure 2, Table S2).

No differences in methane production (g/d) were found among cows in the three lactation periods, with all averaging approximately 400 g/d in our study (Figure 2, Table S2). The methane production patterns observed in this study were similar to those measured by Pszczola et al. [32] using a sniffer, predicted by de Haas et al. [33] using feed intake and by Kandel et al. [34] using mid-infrared spectroscopy, and to those reported by Coppa et al. [35], who found similar methane production between cows in different lactation periods as measured using the GreenFeed system. Nevertheless, in our study, cows in the mid-lactation period had the highest methane yield, methane emissions per kg of fat-corrected milk yield, and methane energy as a percentage of gross energy intake, at 34.50 g/kg·DMI, 31.67 g/kg·FCM, and 10.58%, respectively. Furthermore, methane intensity (g/kg·milk) fluctuates throughout the lactation cycle; early in lactation, before fat- and protein-corrected milk reaches its peak, milk yield increases faster than methane production, leading to an expected decrease in methane intensity [36]. In mid-lactation, as fat- and protein-corrected milk declines while methane production remains stable, methane intensity inherently increases again [36]. High dry matter intake is typically associated with a rapid passage rate of particulate matter from the rumen to the lower digestive tract, which restricts ruminal degradation and consequently leads to lower methane yield [37]. As the rumen progressively adapts to dry matter intake changes, the passage rate may slow down from early to late lactation, enabling more complete digestion and driving a progressive increase in methane yield [36]. Therefore, the highest methane yield and intensity observed in mid-lactation dairy cows may be attributed to their lower dry matter intake and milk production, combined with the highest dietary fiber digestibility, which provided more hydrogen and carbon dioxide for methanogenesis in our experiment. These differences in methane yield and intensity at different lactation periods were also reported by Coppa et al. [35], which may be attributable to changes in dry matter intake and milk production due to the physiological evolution of lactation. Although the methane production of cows in the early lactation period was similar to that of the other two periods, the highest methane emission per kg of metabolic weight was found in early-lactation cows, at 3.37 g/kg⋅BW^0.75^. This is primarily because early-lactation cows tended to have lower body weights due to their pregnancy status (not pregnant or recently conceived) and negative energy balance, which mathematically results in higher methane emissions when normalized by metabolic weight.

Inconsistent with these findings, a previous study reported that methane emissions of Holstein cows, as measured by a respiration chamber and the GreenFeed system, showed that methane production in the mid-lactation (wk 14) and late-lactation (wk 42) periods was significantly higher than that in early lactation (wk 3) [38]. Furthermore, methane yield measured by the respiration chamber and the Greenfeed system was lower in the mid-lactation period than in the late-lactation period [38]. In the experiment conducted by Rischewski et al., the days in milk of cows in the early lactation stage were only the 3rd week, and the reduction in feed intake caused by changes in gastrointestinal tract position brought about by calving did not recover, resulting in low methane production at this stage [38]. Although the ranking of methane yield in mid-lactation in this experiment was different from that reported by Rischewski et al. [38], methane yield was negatively correlated with dry matter intake in both experiments. These again confirm that feed intake is the primary driver of enteric methane emissions.

### 2.3. Methane Emissions from Dry Cows of Different Parities

**Figure 3:**
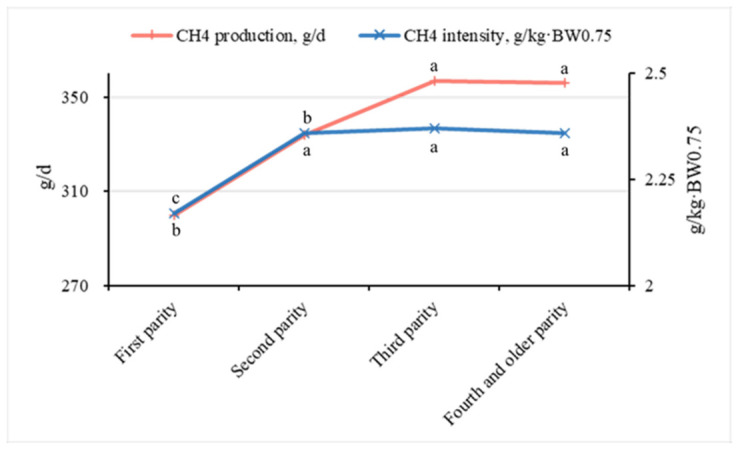
Methane emissions from dry cows of different parities. Within the same indicator, different letters indicate significant differences (*p* < 0.05). *n* = 12. CH_4_: methane; BW^0.75^: metabolic weight.

Dry cows are important members of dairy cattle herds. We measured methane emissions from dry Holstein cows of 1st, 2nd, 3rd, and 4th and older parities, and measured their rumen fermentation parameters. Twelve cows in each group (parity) were fed the same diet containing 85% forage and 15% concentrate (Table S10). The experimental period lasted 15 days, consisting of a 5-day adaptation period followed by a 10-day measurement period. After the adaptation period, the methane emissions and body weight of each cow were measured according to the Jia’s method [39,40]. Notably, this study used the GreenFeed system, the latest technology for directly measuring methane emissions from ruminants, for the first time in China. We observed that the methane production of dry cows was approximately 335 g/d, and the methane intensity was approximately 2.31 g/kg·BW0.75 (Figure 3, Table S3).

The methane production of dry cows increased from 300 to 356 g/d with parity, and that of dry cows of 2ed and older parities was significantly higher than that of 1st parity cows. Similarly, Coppa et al. [35] also observed that the methane production of multiparous cows was significantly higher than that of primiparous cows. Referring to the method of Jia et al. [41] and Zhang et al. [42], we examined the rumen fermentation parameters of dry cows and found no differences in the rumen pH value, total volatile fatty acid content, acetate ratio, and propionate ratio among cows of different parities. Feed intake directly determines methane production from ruminants [43], so this difference in methane production may be caused by the lower dry matter intake in primiparous cows compared to multiparous cows [44]. In addition, the body weight of dry cows exhibited a similar trend to methane production, as did methane emissions per kg of metabolic weight. This result may be attributed to the fact that dry matter intake in dairy cows increases significantly with age and is positively correlated with body weight [30]. A previous study also showed that methane production or methane emissions per unit of dry matter intake, body weight, and milk production were significantly correlated with age [30]. However, the results of the previous study were partly inconsistent with ours, as the methane emissions of cows reached a maximum at 2000 days of age and began to decline in that study [30], whereas our experiment found that the methane emissions of cows reached a maximum at 1800 days (3rd parity) and did not show a downward trend. Interestingly, dry matter intake of cows in the previous study did not begin to decline after 2000 days of age, but was positively correlated with age [30]. However, changes in fiber digestibility with age were similar to methane emissions, which could explain why methane emissions did not respond to age-related changes in dry matter intake [27]. The lower methane emissions in previous studies were mainly concentrated in the 5th to 7th parities [30]. Since it is common for dairy farms in China to eliminate older cows, only 3 cows with a parity greater than 4 were collected in our experiment, which may lead to the lack of a downward methane trend in our experiment.

The results of the above experiments demonstrated that the methane production of lactating and dry cows was 137.7% and 110.7% higher than that of heifers, respectively. Consistent with our findings, the methane emissions of mature animals were higher than those of young animals, which was found in studies of cattle, sheep and red deer [45,46,47]. The IPCC uses the fixed methane conversion factor (methane energy as a percentage of gross energy intake, 6.5%) to calculate the enteric methane emissions from lactating dairy cows and heifers in China [48]. The methane conversion factors for heifers and lactating cows in our experiments were approximately 6.6% and 5.0%, respectively. If the methane emission factors that are recommended by the IPCC are used to estimate methane emissions from dairy cows in China, it will inevitably lead cause errors.

## 3. Methane Emissions from Holstein Dairy Cattle at Different Diet Structures

Dietary nutrient composition, especially fiber, is an important factor affecting ruminant methane emissions; consequently, we investigated the effect of diets with different NDF/NFC ratios on methane emissions from heifers and lactating cows. Methane emissions from Holstein dairy cattle were measured using the sulfur hexafluoride tracer technique.

### 3.1. Effects of Diets with Different NDF/NFC Ratios on Methane Emissions from Heifers

**Figure 4:**
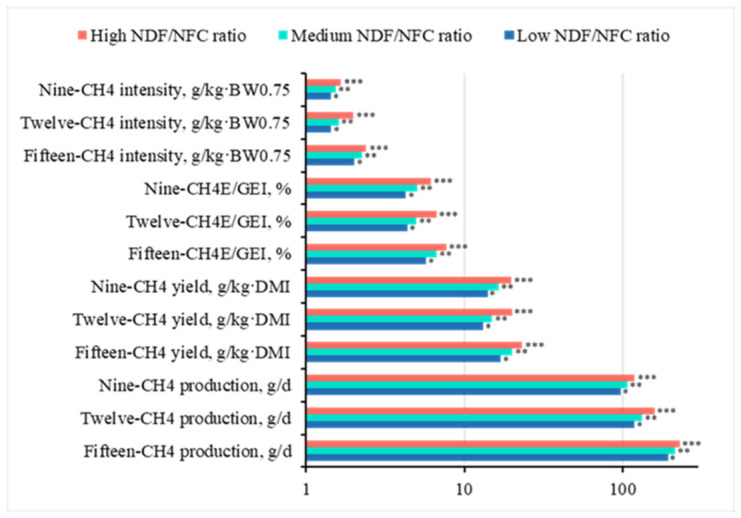
Methane emissions from heifers that were fed diets with different NDF/NFC ratios. Within the same indicator, different numbers of asterisks (*) between groups indicate significant differences (*p* < 0.05). *n* = 5. Nine: 9-month-old heifers; Twelve: 12-month-old heifers; Fifteen: 15-month-old heifers; CH_4_: methane; BW^0.75^: metabolic weight; CH_4_E: methane energy; GEI: gross energy intake; DMI: dry matter intake.

In this experiment, methane emissions were measured from 9-, 12-, and 15-month-old heifers. Forty-five heifers per age group were divided into three groups and fed diets with different NDF/NFC ratios of 0.60, 0.75, and 0.90 (Table S11). Following a 14-day adaptation period to the experimental diets, methane emissions from cows were measured for 14 consecutive days using the sulfur hexafluoride tracer technique. Methane emissions of Holstein heifers were measured utilizing the sulfur hexafluoride tracer technique [19,20]. On the final day of the experimental period, the body weight of the heifers was recorded using an electronic weighbridge prior to the morning feeding. The methane emissions of heifers increased gradually with increasing NDF/NFC ratios (for example, the medium NDF/NFC ratio groups showed an increase in methane production by 9.7% to 12.0%, and the high NDF/NFC ratio groups by 18.5% to 34.1% compared with the low NDF/NFC ratio groups), as did the methane yield, methane intensity (g/kg·BW0.75), and methane energy as a percentage of gross energy intake (Figure 4, Table S4).

The most pronounced differences in methane emissions between the medium or high NDF/NFC ratio and low NDF/NFC ratio diets were observed in 12-month-old heifers when compared to the 9- and 15-month-old cohorts in our study. Previous studies have demonstrated that multiparous cows exhibit greater variation in methane yield and emission intensity (methane per unit of milk yield) compared to primiparous cows, a discrepancy likely driven by higher dry matter intake and milk production in multiparous animals [44]. During the heifer stage, the primary physiological objective is skeletal and muscular development, manifesting as body weight gain. Body weight has emerged as a key variable in optimal models for predicting enteric methane production in sheep [43], and mitigating methanogenesis could partition more metabolizable energy toward tissue accretion in heifers [49]. Consequently, elevated feed intake and rapid weight gain may drive substantial variation in enteric methane emissions among growing heifers. Interestingly, despite these variations, the dry matter intake and average daily gain, which are factors intrinsically linked to methanogenesis and energy utilization, were at intermediate levels in the 12-month-old group compared to the 9- and 15-month-old cohorts. This phenomenon aligns with the fundamental growth trajectories and metabolic dynamics of developing ruminants.

### 3.2. Effects of Diets with Different NDF/NFC Ratios on Methane Emissions from Lactating Cows

**Figure 5:**
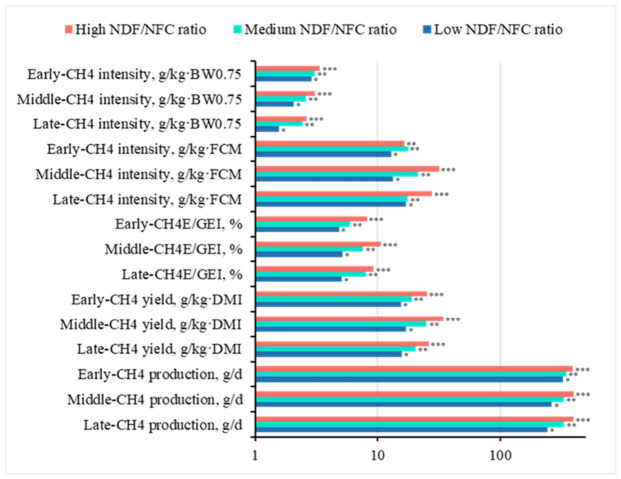
Methane emissions from lactating cows that were fed diets with different NDF/NFC ratios. Within the same indicator, different numbers of asterisks (*) between groups indicate significant differences (*p* < 0.05). *n* = 4. Early: early-lactation cows; Middle: mid-lactation cows; Late: late-lactation cows; CH_4_: methane; BW^0.75^: metabolic weight; FCM: fat-corrected milk; CH_4_E: methane energy; GEI: gross energy intake; DMI: dry matter intake.

A total of 36 cows from early, mid and late lactation periods were selected as experimental animals, with 12 cows in each lactation period. Within each period, cows at each lactation period were divided into three groups and fed diets with different NDF/NFC ratios (Table S12). Cows in the three groups in early lactation period were fed diets with NDF/NFC ratios of 1.14, 1.30 and 1.55, respectively. Similarly to the early lactation period, the NDF/NFC ratios in mid-lactation period were 1.44, 1.65, and 1.82, and the NDF/NFC ratios in the late lactation period were 1.52, 1.96, and 2.10. Following a 14-day to the experimental diets, methane emissions from cows were measured for 14 consecutive days using the sulfur hexafluoride tracer technique. The digestion trial followed the same methodology as the aforementioned heifer trial. Methane emissions of Holstein heifers were measured utilizing the sulfur hexafluoride tracer technique [20,21]. The measurement of lactational performance in dairy cows was based on the method described by Dong et al. [31]. After measuring methane emissions from the cows, the results showed that the methane production, methane yield, methane intensity (g/kg·milk or g/kg·BW0.75), and methane energy as a percentage of gross energy intake of the cows were positively correlated with the NDF/NFC ratio (Figure 5, Table S5).

Using these data, we developed methane prediction equations and evaluated their performance (Tables S6 and S7). Compared to the globally utilized IPCC (2006) [50], Yan et al. (2000) [51], and Niu et al. (2018) [22] prediction models, the enteric methane emission prediction equations (Equations (1)–(5)) established in this study demonstrate significant advantages in local adaptability and predictive accuracy. Primarily, the international universal models exhibit a severe overestimation of emissions when applied to the Chinese Holstein dairy cow population. For instance, while the measured average annual methane emission is approximately 115.3 kg/cow, the estimates derived from Equations (1) and (2) in this study are 116.5 and 115.6 kg/cow, respectively, which closely align with the actual observations. In contrast, the Yan and Niu models yielded significantly inflated estimates of 120.9 and 121.5 kg/cow, respectively. Furthermore, the core equations developed in this research substantially reduce the overall prediction error. Specifically, Equations (1) and (2), which are based on dry matter intake and NDF intake, successfully lower the root mean square prediction error (RMSPE) to 17.7% and 18.2%, respectively. These values are markedly superior to those of mainstream international models such as the IPCC (36.4%), Yan (31.1%), and Niu (27.5–34.7%). Additionally, an analysis of the error decomposition matrix reveals a fundamental optimization in the sources of error within the new models. A considerable proportion of the error in the international models originates from the error in central tendency (ECT), which reflects systemic bias (IPCC: 21.5%, Yan: 39.8%, Niu: 12.7–51.5%). This exposes their limitations stemming from the disregard of geographical variations, animal physiological conditions, and underlying dietary substrates (e.g., high-quality forage systems in Western countries). Conversely, the ECT of the newly developed core models (such as Equations (1) and (5)) approaches zero, effectively mitigating this systemic discrepancy. This indicates that their internal mathematical logic perfectly captures the metabolic characteristics of typical Chinese dairy diets, which are rich in corn silage and various agricultural by-products, thereby providing a robust scientific foundation for more precise, localized greenhouse gas accounting.

There have been many studies reporting the relationship between dietary fiber and enteric methane emissions; for instance, increases in the forage ratio generally contributed to increases in methane emissions [1,52]; NDF content appeared in the best model of methane production in sheep [43], with a positive correlation between these two variables [53,54]; and fiber-rich diets produced more methane than did lipid- or starch-rich diets in cows [55]. Our results were in line with these studies, which may be because dietary NDF produced more methane in the rumen than did faster-fermenting carbohydrates [56]. The ruminal fermentation pathways for acetate and propionate exert fundamentally different effects on hydrogen dynamics. The synthesis of acetate releases metabolic hydrogen, providing the primary substrate for methanogenesis, whereas propionate production serves as a competitive hydrogen sink. Consequently, diets rich in fibrous carbohydrates promote acetate-type fermentation and increase hydrogen availability, whereas an increased supply of NFC favors propionate-type fermentation, thereby restricting the hydrogen pool available for methane production. Therefore, variations in the carbohydrate distribution in the diet influence methanogenesis, i.e., higher NDF/NFC ratios result in higher methane emissions. Conversely, reducing NDF/NFC ratios actively shifts rumen fermentation away from hydrogen generation toward propionate and butyrate production, thereby significantly decreasing overall methane emissions [57]. Lignin, as a component of NDF, cannot be fermented and degraded in the rumen [43]. It is important to note that increases in lignin increased only the NDF content of the diet but did not cause greater enteric methane emissions [43].

Mitigating enteric methanogenesis may provide cows with additional ME that can be used for production purposes [48]. In other words, reducing methane production relative to milk yield is the main goal of research on methane abatement in lactating cows. Increased concentrate content in dairy cow diets generally leads to increased milk production and reduced methane production by intensifying propionate-type fermentation and reducing the populations of protozoa, cellulolytic bacteria and methanogens (*Methanobrevibacter smithii*, *Methanosphaera stadtmanae*) in the rumen [30,58,59]. In agreement with these findings, the increase in NFC content was beneficial in reducing the methane intensity (methane emissions per kg of milk yield) in our study. In addition to influencing enteric methane emissions, increased dietary NDF content has been shown to elevate the excretion of fecal dry matter and total nitrogen. For instance, Lingen et al. [43], through a quantitative joint evaluation of sheep fed diverse forage-based diets, demonstrated that as dietary NDF intake increased, the reduced fiber digestibility led to a higher proportion of dry matter and nitrogen being excreted in the feces. Therefore, even when considering the emission of greenhouse gases from feces, the reduction in dietary NDF content was beneficial for reducing the environmental impact of the dairy industry.

While increased NFC content in the diet was beneficial for methane abatement, the production of concentrate produces more greenhouse gases than does forage, which offsets its mitigation effect on methane [59,60]. Nevertheless, providing highly digestible feed, such as energy-dense grains, remains a well-researched option for mitigating enteric methane [61]. While this practice may offer limited additional benefits in highly developed animal production systems, it holds considerable mitigation potential in developing countries. Assuming a 10% increase in the digestibility of the basal diet, the technical mitigation potential of this practice could reach approximately 0.68 Gt tons of carbon dioxide equivalents per year globally, although its actual economic mitigation potential depends heavily on adoption rates, historically ranging closer to 0.12 to 0.15 Gt tons [61,62,63]. In addition, the increase in the use of concentrates will also exacerbate the problem of food competition between humans and livestock [64]. The increase in other greenhouse gases caused by increasing the proportion of NFC is also a problem we face. Using cow manure for feed cultivation near cattle farms may be an effective way to mitigate these additional greenhouse gas emissions.

The success of a methane reduction strategy depends on a large and accurate database of farm animal methane emissions. Through these several experiments, we found that lactating cows had the highest methane emissions, heifers had the lowest, and dry cows exhibited intermediate levels. Therefore, we hypothesize that methane abatement strategies developed for lactating cows will yield the largest reductions. However, heifers and dry cows cannot be ignored. A study has shown that heifers within 3 years of first calving release more enteric methane emissions than heifers within 2 years of first calving, and 50% of the emissions from birth to calving in 3-year-old heifers were emitted within 24 to 36 months [65]. The dairy industry needs to focus on heifer conception rates to reduce investment costs and methane emissions. Diets that are fed to dry cows and heifers on farms generally have higher NDF ratios, and reducing this ratio will abate methane emissions if a balance is maintained between the cattle’s nutrient requirements, ration costs and abatement effects. At present, research on methane mitigation strategies mainly focuses on breeding measures, farm management practices (e.g., proper barn ventilation and air filtration), and nutritional strategies, including the application of various microbial feed additives [66,67]. If these multi-dimensional mitigation strategies can be combined and applied, the livestock industry will achieve more effective and holistic methane abatement [68]. Meanwhile, evaluating the synergistic effects of these combined approaches remains a significant challenge for future research.

## 4. Conclusions and Future Research

We obtained the enteric methane emissions of dairy cattle across their primary physiological stages, namely, the growth, lactation, and dry stages, with the highest methane emissions from lactating cows, the lowest from heifers, and intermediate levels from dry cows. Based on these several experiments, we determined that methane production in heifers increased with age, methane production in lactating cows did not change during lactation, and methane production in dry cows reached a maximum at the third parity and tended to stabilize. The methane emissions of dairy cattle were affected by the NDF/NFC ratios in the diet. Compared to the model established based on our experiment, models from other countries cannot accurately predict the methane emissions of dairy cows in China. A methane emissions database is an important basis for calculating the methane emissions of a region and formulating methane mitigation strategies, which need to be established from a comprehensive and large amount of basic data. Therefore, we measured methane emissions from heifers, lactating cows, and dry cows and investigated the effect of dietary fiber levels on methane emissions from dairy cattle. However, we fully acknowledge that generating robust, country-specific methane emission factors inherently requires empirical measurements across a much larger number of animals than what was evaluated in our specific cohorts. Therefore, it is also necessary to accumulate methane emissions data from large-scale populations of dairy cattle in other regions, seasons, and feeding conditions to supplement and improve China’s methane emissions database for dairy cattle.

## Data Availability

Data is available at a reasonable request to the corresponding authors.

## Supplementary Materials

The following supporting information can be downloaded at: https://www.mdpi.com/article/10.3390/ani16111684/s1. Table S1: Methane emissions and apparent nutrient digestibility in heifers at different ages; Table S2: Lactation performance, methane emissions, and apparent nutrient digestibility in lactating cows at different lactation periods; Table S3: Methane emissions and rumen fermentation parameters in dry cows of different parities; Table S4: Methane emissions from heifers that were fed diets with different NDF/NFC ratios; Table S5: Methane emissions from lactating cows that were fed diets with different NDF/NFC ratios; Table S6: Prediction equations of enteric methane emissions for Chinese lactating Holstein dairy cows and extant methane prediction equations; Table S7: Mean square prediction error analysis using developed and extant methane prediction equations; Table S8: Composition and nutrient levels of basal diets for Holstein heifers (DM basis %); Table S9: Composition and nutrient levels of basal diets for Holstein lactating cows (DM basis %); Table S10: Composition and nutrient levels of basal diets for Holstein dry cows (DM basis %); Table S11: Composition and nutrient levels of basal diets for Holstein heifers with different NDF/NFC ratios (DM basis %); Table S12: Composition and nutrient levels of basal diets for Holstein lactating cows with different NDF/NFC ratios (DM basis %).
